# Dental status, visits, and functional ability and dietary intake of elderly in Israel

**DOI:** 10.1186/s13584-018-0252-x

**Published:** 2018-12-10

**Authors:** L. Natapov, D. Kushnir, R. Goldsmith, R. Dichtiar, S. P. Zusman

**Affiliations:** 1 Dental Health Division, Ministry of Health, 39 Yirmiyahu Street, 9446724 Jerusalem, Israel; 2 Nutrition Department, Ministry of Health, Jerusalem, Israel; 3 Israel Center for Disease Control, Ministry of Health, Jerusalem, Israel

**Keywords:** Elderly, Dental status, Subjective oral health, Dietary intake, Mabat Zahav

## Abstract

**Background:**

Epidemiological studies have shown deterioration in dental health accompanying the ageing process. Tooth loss increases with age. Chewing ability is closely correlated with number of natural teeth present: there is a threshold of 20–21 teeth, below which chewing ability declines. The government of Israel is currently considering adding dental treatment for elderly to the basket of services of the National Health Insurance Law. Information on the influence of elderly’s dental health on nutrition and general health status can contribute to the decision making process.

**Methods:**

Secondary analysis of data collected on a subsample (*N* = 1776) of the cross-sectional Mabat Zahav - National Health and Nutrition Survey of the Elderly was done. Intakes of energy, fiber, protein, fruits and vegetables, associations with dental visits, dentures presence and functional ability were analyzed. Linear regression adjusted for confounders was performed.

**Results:**

Statistically significant differences in dietary intake of energy, fiber, protein and vegetables were found between elderly who visited a dentist in the last year and those who did not. Elderly who possessed dentures had lower dietary intakes than their dentate counterparts. Elderly with functional problems such as impaired chewing had worse dietary intakes than the others. This was so after controlling for education, degree of interest in the relationship between nutrition and health and reading the nutrition label.

**Conclusions:**

The findings in our study suggest that those who visited a dentist in the last year, had natural teeth and no denture/s and reported no chewing problems had better dietary intake. The results emphasize the importance of maintaining adequate dental health, preserving natural teeth and regular dental visits in the elderly to assure adequate nutrient status in this age group.

## Background

Large–scale national surveys, including the USDA Food Consumption Surveys and National Health and Nutrition Examination Surveys in the U.S. and the National Diet and Nutrition Survey in Britain found inadequate intake of several nutrients in a high percentage of older people [1, 2].

The adequate nutrition is very important for the elderly’s health. Impaired nutrition has been associated with a variety of morbid conditions including cancer, heart disease, stroke and dementia in persons over the age 65 [3].

It is a well-known fact that aging is associated with having fewer teeth. Many epidemiological studies in oral health have clearly shown deterioration in dental health accompanying the ageing process [4].Older people often have increasingly poor oral hygiene, higher levels of plaque and calculus together with a higher prevalence of periodontal disease [5, 6].

Chewing ability is closely correlated with number of natural teeth present: there is a threshold of 20–21 teeth, below which it declines [7]. Removable partial denture in the lower jaw which replaces molar teeth does not improve oral function in terms of chewing ability [8].

Loss of teeth also alters older people’s selection of foods. They tend to choose softer and more easily chewed foods which are often lower in fiber and less nutrient-dense [9].

It has been noted [10] that those with severe tooth loss had the lowest dietary quality and avoided the most foods. Poor masticatory performance has been associated with lower frequency of consumption of raw carrots and fresh fruits and vegetables [11]. Joshipura et al. [12] found that edentulous health professionals consumed fewer vegetables, less dietary fiber and carotene and more cholesterol, saturated fats and calories than people with 25 or more teeth.

The percentage of elderly in Israel has doubled since the 1950s and is expected to reach 15% by 2035 [13]. The Israeli elderly are of diverse places of birth, with more than 100 countries of origin. The percentage of Israeli born elderly is rising too. This cultural diversity is unique and may have an effect on dietary pattern and intake. There are no data on the impact of dental status and visits on dietary intake among this population in Israel. It is optimal if public policy decisions are supported by local data, especially for mobilizing support to promote them, therefore it is of value to find whether there is an association between oral health and dietary intake in Israel.

The present study is the first attempt to investigate this impact at a national level in Israel. It is based on data collected from those interviewed for the very first National Health and Nutrition Survey of the Elderly (Mabat Zahav), carried out in 2005–6 by the Ministry of Health. The aim of the Mabat Zahav survey was to collect basic data on health (including oral health) and nutritional status of a national random sample of elderly, aged 65 and over, in Israel so as to provide updated and reliable data for developing food and nutrition policy.

The aim of our study is to assess the impact of dental status and visits on dietary intake of the Israeli elderly.

## Methods

One thousand eight hundred fifty adults, age 65 and over, were interviewed in their homes, after signing an informed consent form. Interviewers were specially trained in the questionnaire administration and in the protocols for carrying out anthropometric measurements. A full description of the materials and methods of the Mabat Zahav survey is reported elsewhere [14].

### The sampling frame

Sixty-five year–old and over people, living in the community (their own homes or sheltered houses), insured medically with two (Clalit and Maccabi) of the four health funds, were included in the sample. These two health funds cover 86.3% of the population age 65 and over. The inclusion criteria were: Israeli citizens with place of residence defined as Israel. Exclusion criteria: elderly not living in the community and new immigrants.

### The questionnaire

A face-to-face personal interview was conducted in the interviewees’ homes using a structured questionnaire. The questionnaire was written in Hebrew and translated into Russian, Arabic and English. The questionnaire included demographic details such as gender, age, population group, education years and attainment and economic status, questions on health status, dental health, dietary intake and eating habits and associated health behaviors.

In order to ensure that answers given could be relied upon i.e. that the responder was cognitively aware, a cognitive status (Mini Mental State Examination (MMSE)) [15] questionnaire was also included. Questionnaires of those scoring less than 17 were not included in the final data analyses and dataset.

In order to estimate the dietary intake of the population, the 24 h dietary recall method was used [16], whereby interviewees were asked to recall all that they had eaten and drunk in the 24 h period that preceded the interview. The validity of this method has been reported extensively.

The trained interviewers used aids, including the specially developed “Israel Food and Food Quantities Guide”, to improve accuracy of recalls. All data was entered into the Tzameret computerized Dietary Analysis program.

The questionnaire also included 7 questions on subjective dental health status: a question about subjective dental health, one about last visit to dentist, a question about owning removable (partial or full) dentures and one about using them, about masticatory problems, about consuming soft food because of inability to chew and if yes, what kind of soft food.

Additionally, the questionnaire included the 14 questions of OHIP14. The results of the OHIP14 were reported elsewhere [17].

From the people that agreed, 299 were clinically examined in their homes according to WHO Oral Health Survey methods ed.4 [18] to objectify their dentate status. The non-dentures group had on average 20 teeth and 4.1 posterior occluding pairs of teeth, i.e. a functional shortened dental arch [19].

### Statistical analysis

Statistical analysis was carried out in the SAS program (version 9.1.3). Statistical significance of continuous variables was assessed using the Students’ t-Test (assuming normal distribution) and categorical variables using the Chi square test. The Wilcoxon Mann-Whitney two sample test was used where distributions were not normal.

Multiple linear regressions were performed to assess the association between the dependent variables: intake for energy, protein, fiber, fruits and vegetables and the independent ones: visit to the dentist, dentures presence and chewing problems while adjusting for confounders: education, interest in association between nutrition and health and reading nutrition labels.

## Results

A total of 1852 elderly were included in the sample. Questionnaires were completely filled for 1776, and those were analyzed.

Mean energy intake was 1527 kcal/day for those who visited a dentist within the last year, compared to 1389 kcal/day for those who did not. Four nutrient variables – energy, fiber, protein and vegetables were found to be significantly higher for those who visit the dentist more frequently (Table 1) in multivariate analysis while for fruits the difference was not statistically significant.

**Table 1:** Daily dietary intake of elderly by dental visit

		N	Energy Mean (sd)	Fiber Mean (sd)	Protein Mean(sd)	Fruit Mean(sd)	Vegetables Mean(sd)
			Kilocalories	Grams	serves	serves	serves
Visit to Dentist	Within the past year	822	1527(595)	18.6(9)	3.6(2.1)	1.7(1.6)	2.5(1.9)
	More than a year ago	953	1389 (576)	15.9(8.6)	3.2(1.9)	1.4(1.4)	2.2(1.9)
	Univariate analysis		*p*<0.0001	*p*<0.0001	*p*<0.0001	*p*<0.001	*p*<0.001
	Linear regression		*p*<0.001	*p*<0.001	*p*<0.001	ns	*p*<0.004

sig = *p*<0.05

Energy mean intake in the no-denture group (had on average 21 teeth and 4.1 posterior occluding pairs) was 1522 kcal/day while it was 1413 kcal/day in the denture group (*p* < 0.006 in multivariate analysis). Elderly who had no dentures had significantly better intakes of 4 out of 5 parameters - energy, fiber, protein and vegetables than the elderly with dentures (Table 2). For fruits the difference was not statistically significant.

**Table 2:** Dietary intake by dental status

		N	Energy Mean(sd)	Fiber Mean(sd)	Protein Mean(sd)	Fruit Mean(sd)	Vegetables Mean(sd)
			Kilocalories	grams	serves	serves	serves
Dentures	No	640	1522 (599)	18.6(8.9)	3.6(2.1)	1.6(1.5)	2.6(2.0)
	Yes, one or two jaws	1140	1413 (579)	16.3 (8.7)	3.3(1.9)	1.5(1.5)	2.1(1.8)
	Univariate analysis		*p*<0.001	*p*<0.0001	*p*<0.005	ns	*p*<0.0001
	Linear regression		*p*<0.006	*p*<0.001	*p*<0.0014	ns	*p*<0.0004

sig = *p*<0.05

Table 3 shows that significant differences in dietary intake were related to reported chewing problems. Chewing problems were associated with lower intakes of 4 variables, and were statistically significant. For fruits the difference was again not statistically significant (*p* = 0.068).

**Table 3:** Dietary intake by reported chewing problems

J5		N	Energy Mean(sd)	Fiber Mean(sd)	Protein Mean(sd)	Fruit Mean(sd)	Vegetables Mean(sd)
			Kilocalories	grams	serves	serves	serves
Chewing problems	Yes	384	1355( 599)	15.3(8.4)	3.2 (2)	1.3 (1.4)	2.0 (1.9)
	No	1396	1480(583)	17.6(8.9)	3.5(2)	1.6 (1.5)	2.4(1.9)
	Univariate analysis		p<0.05	p<0.0001	p<0.05	p<0.05	p<0.0001
	Linear regression		p<0.005	p<0.007	p<0.01	ns	p<0.02

## Discussion

To assess the representativeness of our sample, a comparison was done between those interviewed and those who were not (reported in detail elsewhere [14]. Our sample was similar to the Mabat Zahav sample with respect to sex, age and population group distribution, with minor differences in education and functional limitations reported.

This did not substantively affect the representativeness of the sample.

Data collected in the Mabat Zahav survey was used to estimate, with a greater degree of certainty, the need for health services to ensure an appropriate distribution of services for this age group, to facilitate the provision of optimal nutritional services to those elderly living in the community, thereby leading to an improvement in their quality of life.

The number of elderly in the Israeli population is rising and this study is the first and until now the only large national population-based survey that allows us to assess the nutrient intake in relation to the oral health of the Israeli elderly. Being the very first and only such data, it is still relevant despite the time that passed since its collection. Nothing significant of relevance to this study in total energy or nutrients intake has changed for the elderly in the past decade [20].

Adequate energy intake may have a crucial role in maintaining a healthy Body Mass Index. Sheiham et al. found that older people in Britain with more than 20 teeth are more likely to have a normal Body Mass Index [21].

Identifying the elderly with compromised dietary intake is important to prevent various comorbidities in this age group. Evaluation of dietary needs is an important skill for dentists and physicians who care for elderly patients [22].

The ability to bite and chew is considered to be very important in the elderly [21].

Tooth loss impairs masticatory ability. Tooth loss is also associated with changes in foods preference and nutritional deficiency in older people.

Research studies have reported that tooth loss impairs masticatory efficiency even after replacement with dentures [23].

Dental status was the only oral clinical variable assessed in this study. The elderly with natural teeth have better dietary intakes than the elderly with dentures. The significant statistical difference was found in intake of fibers, energy, protein and vegetables. Our results support observations of Joshipura et al. [12] who found that loss of natural teeth reduces dietary quality and nutrient intake and emphasizes the importance of preserving natural teeth and adequate masticatory function which the present study confirms.

The fact that older adults are maintaining their natural teeth into their later years has been presented in contemporary epidemiological research [24].

Our study emphasized the importance of regular visits to achieve the adequate nutrient intake in this age group. The elderly who reported dental visit in the last year had significantly better results for all 4 parameters of the dietary intake.

Lack of statistical association to fruit consumption may be explained by wide availability of fruits in our country all around the year in wide variety of textures.

In Israel dental care for the elderly is not yet a part of the National Health Insurance. Universal access and elimination of the barriers for dental treatment are important for preservation of natural teeth in elder age. The upcoming comprehensive national reform in dental care for elders must emphasize the importance of preventive and restorative dental treatment in order to prevent tooth loss and keep natural dentition in the elder age.

The Israeli dental system underwent substantial changes in the last decade [25]. Dental care for children was included in the National Health Insurance law. The entitlement age for this dental care is expected to reach 18 within the next year. The elderly are the next age group to be included in the law, an issue that is discussed and debated nowadays. All additional data like the current article, can aid in the decision making process and the reform implementation. Extending NHI to provide dental care for the elderly will improve their ability to chew and hence also improve their nutritional status, reduce the amount of dental pain they must endure, and in some cases will also improve appearances (important both for self-image and social interactions). Another reason it makes sense to begin the coverage of adults with the elderly is that many of them have limited financial resources and hence face significant financial barriers to care.

It is important to improve access to dental care in Israel for all adults, not just the children and the elderly. Once all adults have good dental care access, fewer of them will reach old age with major oral health problems. Given budgetary constraints and political realities, the extension of National Health Insurance to include dental care will continue to be a step-wise process, as it was the case with children.

Hopefully, the extension of NHI dental coverage to the elderly will not be the last step. The hope is that it will constitute an important stepping stone to help the public and the political leadership appreciate the health, social, economic and political benefits of publicly-financed dental care for all.

### Limitations of this study

The method used to collect information on food and beverage intake- the 24 h dietary recall, is widely used, and is the main method used in surveys such as the NHANES. Thought was given to the possible impact of cognitive decline on reliability of this method in this survey, so the MMSE was incorporated into the questionnaire and those scoring less than 17 were not included in the final analyses. It is possible that there may have been some recall problems with those scoring between 17 and 24.

A further limitation is that there is no clinical record of the dental state of all respondents – nor did we differentiate between the dentures types: full or partial denture.

## Conclusions

The findings in our study suggest that those who visited a dentist in the last year, had natural teeth and no denture/s and experienced no chewing problems had better dietary intake. The results emphasize that functional dentition, preservation of natural teeth and good dental health are essential to assure adequate nutrient status in elderly age group.
